# Supplementary material for: Causal interplay between lactose intolerance and gut microbiota: a combined bidirectional Mendelian randomization and in vivo validation study
Source: Front Nutr. 2026 Jun 1;13:1803337. doi: 10.3389/fnut.2026.1803337 (PMC13265576; doi:10.3389/fnut.2026.1803337)
Supplement: Supplementary file 1 [file Data_Sheet_1.zip › supplementary materials/Forward/leave one out cross validation/ebi-a-GCST90027816.finngen_R12_E4_LACTONAS.pdf]

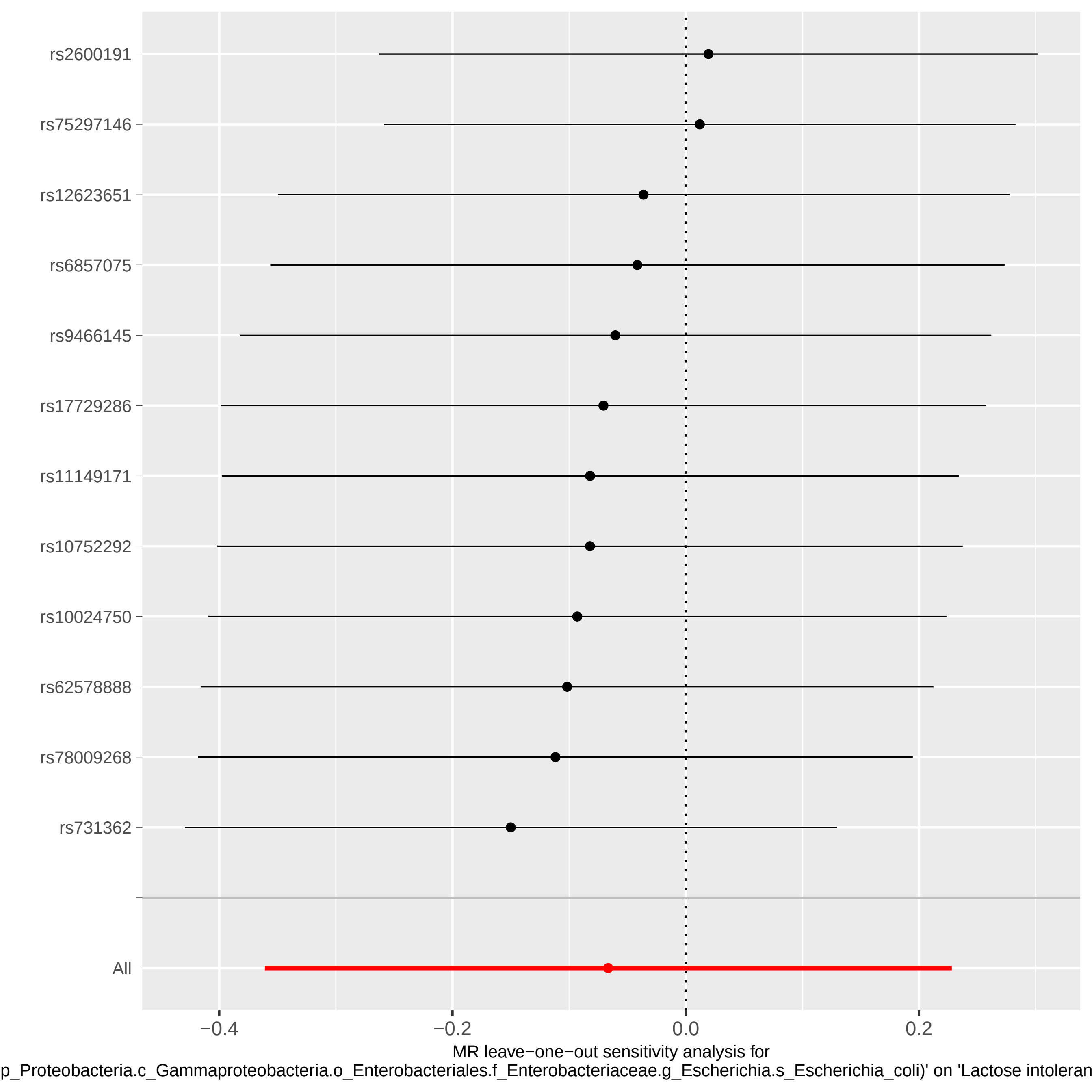
rs2600191
rs75297146
rs12623651
rs6857075
rs9466145
rs17729286
rs11149171
rs10752292
rs10024750
rs62578888
rs78009268
rs731362
All
−0.4
−0.2
0.0
0.2
MR leave−one−out sensitivity analysis for
p_Proteobacteria.c_Gammaproteobacteria.o_Enterobacteriales.f_Enterobacteriaceae.g_Escherichia.s_Escherichia_coli)' on 'Lactose intoleran
